# Global health impact of atmospheric mercury emissions from artisanal and small-scale gold mining

**DOI:** 10.1016/j.isci.2022.104881

**Published:** 2022-08-04

**Authors:** Qiaotong Pang, Jing Gu, Haikun Wang, Yanxu Zhang

**Affiliations:** 1 School of Atmospheric Sciences, Nanjing University, Nanjing, Jiangsu 210023, China

**Keywords:** Environmental chemistry, Environmental health, Pollution

## Abstract

Artisanal and small-scale gold mining (ASGM) is the leading source of mercury (Hg), a global neurotoxin. Past research has focused on the health impacts on miners and nearby residents; here, we estimate the risk for global general populations by employing a comprehensive atmosphere-land-ocean-ecosystem and exposure-risk-valuation model framework. Our results suggest that ASGM sources contribute 12%, 10%, and 0.63% to the atmospheric Hg deposition, plankton methylmercury concentrations, and soil total Hg concentrations at present day, respectively, and cause 5.8×10^5^ points of intelligence quotient decrements and 1,430 deaths for global general populations per year. The monetized global health impact of ASGM ($154 billion) is 1.5 times its local impact and accounts for half of the total revenue of ASGM ($319 billion). A major spatial decoupling between the health impact and economic gains is also revealed, suggesting that intervention measures such as awareness-raising, capacity-building, and technology transfer funded by the Global North are cost-effective.

## Highlights

• Contributions of ASGM to MeHg concentrations in different organisms are calculated
• The global health impact of atmospheric Hg from ASGM is first revealed
• The health impacts and economic gains of ASGM are compared
• Intervention measures for ASGM are proved to be cost-effective

## Graphical abstract

**Figure:**
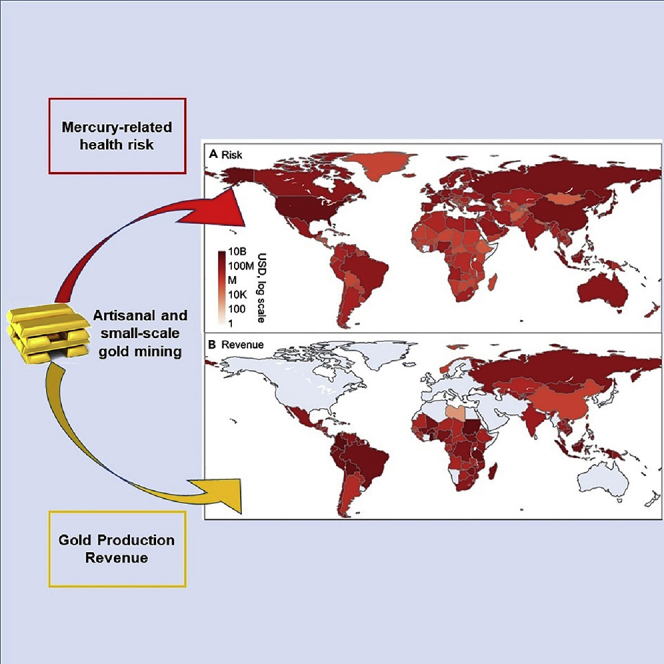

## Introduction

Given the unique nature to form an amalgam with gold, mercury (Hg), a potent neurotoxin associated with neurocognitive deficits in fetuses and cardiovascular diseases in adults (Axelrad et al., 2007; Roman et al., 2011), is widely used by miners in artisanal and small-scale gold mining (ASGM) to subtract gold from low-grade ore all over the world (UNEP, 2018). During the process, Hg is added to a slurry of ore to form a gold-mercury amalgam, which is then burned to evaporate the Hg in an open flame, leaving just the gold (Wade, 2013). With soaring gold prices, ASGM is estimated to produce approximately 600 metric tons of gold per year, accounting for ∼20% of global gold production at the present day, and supports the livelihood of more than 15 million miners in over 70 countries (UNEP, 2018). Meanwhile, more than 1600 metric tons of Hg is consumed per year, with most of them being released into the atmosphere and water environment (Sippl and Selin, 2012). Currently, ASGM becomes the largest source of anthropogenic Hg emissions to the atmosphere globally (675–1000 metric tons yr^−1^, 37% of the total emission), surpassing that of coal-fired power plants (UNEP, 2018). The international Minamata Convention on Mercury that took effect in 2017 has Article 7 specifically directed at this source (UNEP, 2017).

ASGM has heavily elevated the Hg levels in the environment near mining sites and caused significant health impacts on miners and nearby residents (Gerson et al., 2022; Basu et al., 2015; Queipo-Abad et al., 2019; Gibb and O’Leary, 2014; Dong et al., 2015; Rajaee et al., 2015). Due to the long lifetime of Hg in the atmosphere (∼0.5–1 year), the atmospheric emissions from ASGM can also be transported globally and become part of its global biogeochemical cycles (Strode et al., 2009). Atmospheric Hg emissions from ASGM can thus be deposited into the terrestrial and aquatic ecosystems far from its emission regions, where anaerobic organisms can convert them to a more toxic form, methylmercury (MeHg) (Obrist et al., 2018). MeHg is bioaccumulative and can be substantially concentrated in the food webs (Selin, 2009). Therefore, consumption of food (e.g. freshwater fish, seafood, and rice) containing the MeHg transferred from the ASGM emissions may pose a significant health effect on the general populations on a global scale. However, past research has focused on the health impact on miners and nearby residents with that on the global general populations largely unstudied.

In this study, we estimate the health effects of the atmospheric Hg emissions from ASGM on the global general populations, which helps cost-benefit analysis of this activity and to inform potential control policy. A comprehensive atmosphere-land-ocean-ecosystem and exposure-risk-valuation model framework is applied to track the historical ASGM Hg emissions since the 1970s (Zhang et al., 2021). The physical model framework includes 3D atmosphere and ocean models and a 2D land model. In one scenario, we only consider the Hg emissions from ASGM, while the other considers all the anthropogenic and natural emissions. As all the transport, chemistry, and biogeochemical transformation are simulated as linear processes, the ratio of the simulated environmental concentrations can be used to diagnose the contribution of ASGM source to food MeHg levels, and the human exposure and risk are then calculated based on an intake inventory and epidemiology-based dose-response relationships. We analyze the spatial distribution of the ASGM-posed risk for different countries and evaluate the economic value of the health impact (see STAR Methods for details). The policy implications for controlling this source are also discussed.

## Results and discussion

### Environmental Hg levels contributed by ASGM

**Figure 1:**
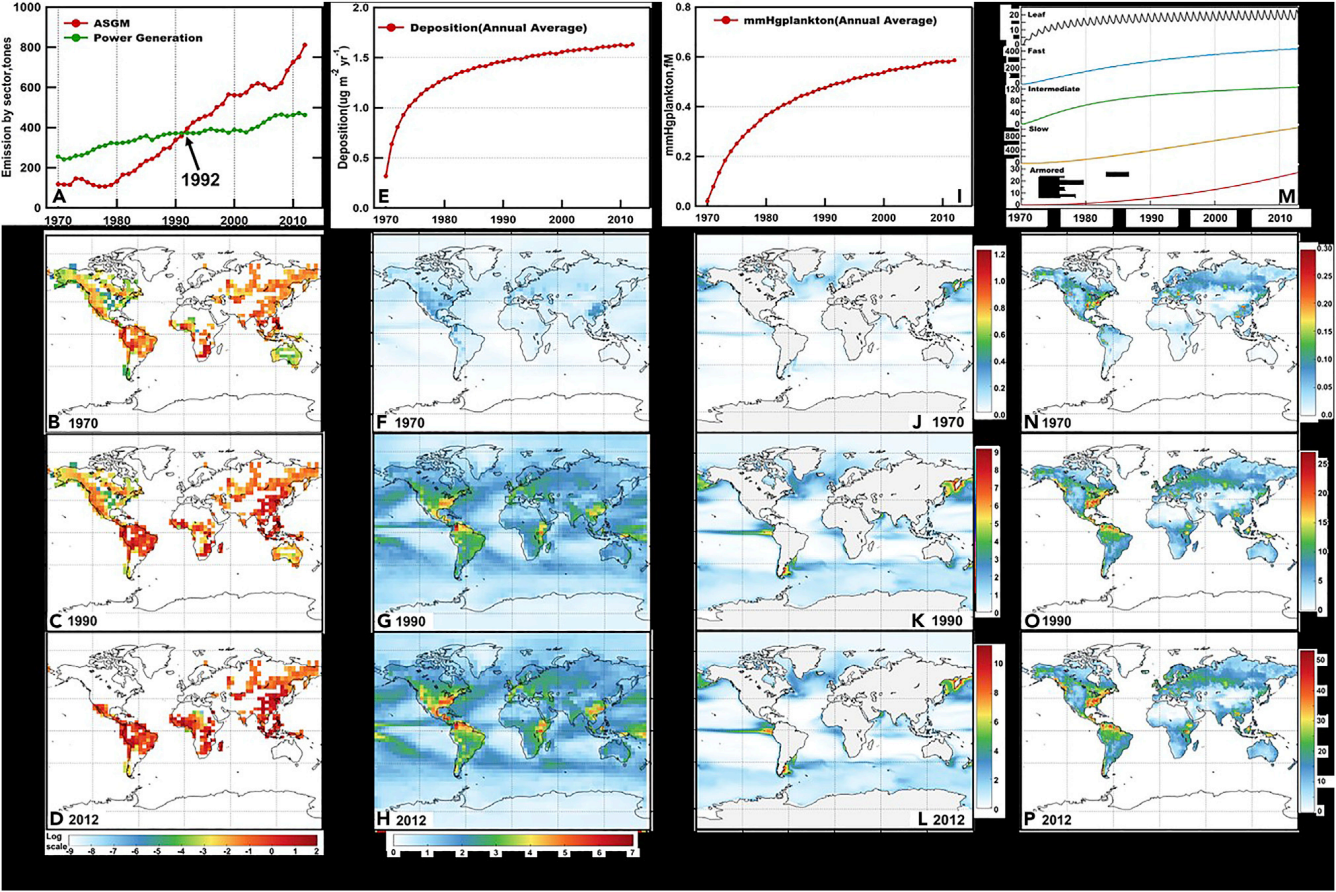
Trends of ASGM Hg emission and its accumulation in the environment (A–D) Hg emissions; (E–H) atmospheric deposition flux; (I–L) plankton MeHg concentrations; (M−P) soil total Hg concentrations. The top panels (A, E, I, and M) show the global trends during 1970–2012, while the lower three show the spatial patterns in 1970, 1990, and 2012. In panel M shown are the Hg masses in the leaf, fast, intermediate, slow, and armored pools.

The accumulated Hg emissions from ASGM during 1970–2012 are 15,400 metric tons with most of the emissions distributed in Sub-Saharan Africa (32%, 4970 tons), South America (29%, 4470 tons), and tropical Asia (17%, 2610 tons), according to the EDGAR inventory (Figures 1A–1D) (Muntean et al., 2014). The transport and transformation of these emissions in the atmosphere, land, ocean, and the exchange between them are modeled by the GEOS-Chem, GTMM, and MITgcm models that are driven by re-analyzed meteorological and ocean physical data. These models are coupled by the NJUCPL and the marine plankton ecosystem dynamics are from the Darwin model (see STAR Methods). By considering only the ASGM source in the model system, and comparing the results with all sources, we can diagnose its contribution to the total environmental burden. The model simulates a global average atmospheric deposition flux of 1.6 μg m^−2^ yr^−1^ in 2012, accounting for 12.2% of the global average at present day. The depositions are the largest near ASGM source regions, such as Columbia and Venezuela in South America, and Kenya and Ethiopia in Sub-Saharan Africa (Figures 1F–1H), where the simulated deposition flux can achieve >5 μg m^−2^ yr^−1^, representing a significant portion of the total deposition there (Figure 1A).

The modeled global average plankton MeHg concentrations are 0.59 fmol L^−1^ (1 fmol = 10^−15^ mol) seawater in 2012, with the largest concentrations in the biological productive tropical and high-latitude oceans (Figures 1J–1l). We find the contribution of the ASGM source to the global average plankton MeHg concentrations are slightly lower (10.4%) than that of atmospheric deposition, reflecting the upwelling of subsurface waters that is less influenced by the ASGM sources (Zhang et al., 2020). The model suggests that the ASGM source has a rather small impact on the soil’s total Hg concentrations (0.63%) due to its large Hg reserve, with a spatial distribution mimicking that of atmospheric deposition (Figures 1N–1P). The model also suggests a non-linear trend for the Hg levels in the environment with increasing ASGM emissions, even though their spatial patterns seem stable. For example, the Hg emissions from ASGM were increased by 6.8 times during 1970–2012, while the modeled associated atmospheric deposition level was only increased by a factor of 5 (Figures 1A–1E). This is caused by the increase of re-emissions of past-deposited Hg from ASGM in the land and oceans.

### Health risks associated with ASGM

**Figure 2:**
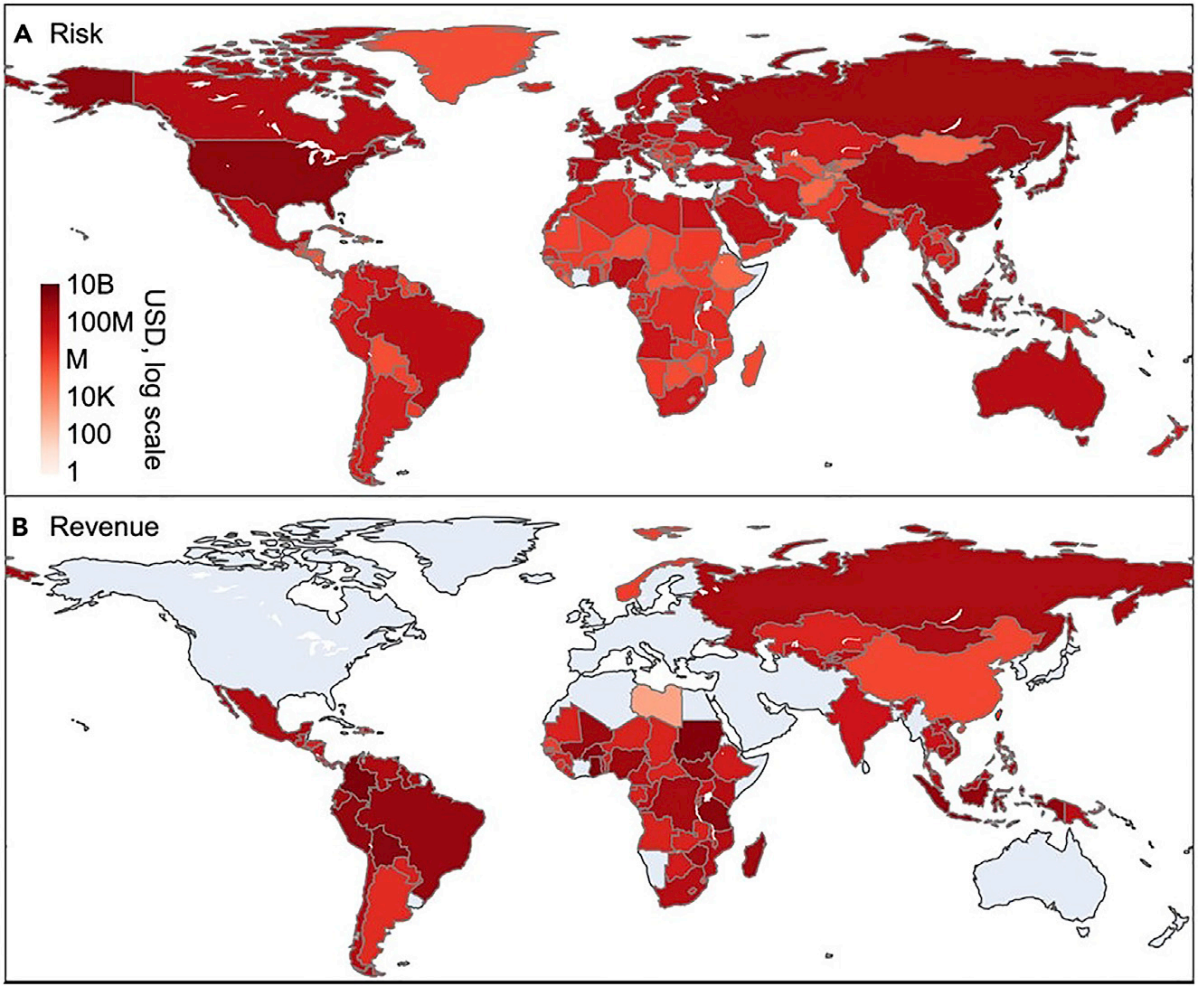
Monetized health impacts and economic revenue of Hg emissions from ASGM in 2012 Economic valuation of the health impact (A) and gain via gold production (B) in 2012 attributable to the Hg emissions from artisanal and small-scale gold mining.

The global health risks for MeHg exposure associated with ASGM sources in 2012 are calculated as 5.8×10^5^ points of intelligence quotient (IQ) decrements and 1,430 deaths, with a global total economic valuation of $7.1 billion per year (US dollar in 2020 value adjusted by purchase power parity, the same hereinafter) (Figures 2 and S2). The health impact is calculated based on a food intake inventory and literature-collected food MeHg concentrations. The contributions of ASGM sources to the food MeHg exposure are estimated based on the modeled environmental Hg levels (Figure S1). We include two endpoints for the health impact: decrease in IQ in newborns and fatal heart attack (FHA) for adults, which are monetized based on the lifelong earning loss (EL) of newborns and the value of statistical life (VSL), respectively (see details in STAR Methods). Overall, we find that 53% of the economic loss associated with ASGM-MeHg exposure to the general population is contributed by IQ decrements ($3.8 billion) with 47% by FHA deaths ($3.4 billion) (Figure S4).

The geographical distribution of the economic loss associated with ASGM (Figure 2A) differs drastically from that of its emissions (Figures 1B–1D) and the associated economic gain via gold production (Figure 2B). In 2012, most of the losses are in Europe (33.2%, 2.4 billion) and Asia (31.7%, $2.3 billion), followed by North America (25.3%, 1.81 billion USD), while Africa (4.3%, $0.31 billion), South America (3.0%, $0.21 billion), and Oceania (2.5%, $0.17 billion) contribute the remaining 9.8% only. The high economic loss in Europe and North America reflects their more developed economies that have higher earning loss with unit IQ loss (Bellanger et al., 2013), and the VSLs are also higher in these regions (Giang and Selin, 2016). The high loss in Asia is contributed by both the large population and the existence of middle- to high-income economies (e.g. China, Japan, and South Korea). For individual countries, the US ($1.6 billion), China ($0.64 billion), Russia ($0.61 billion), and Japan ($0.56 billion) suffer from the largest losses (Figure 2). Countries like France ($0.26 billion), Germany ($0.25 billion), Indonesia ($0.19 billion), Canada ($0.16 billion), and Brazil ($0.12 billion) that have relatively large seafood consumption also have substantial losses (Figure 2).

**Figure 3:**
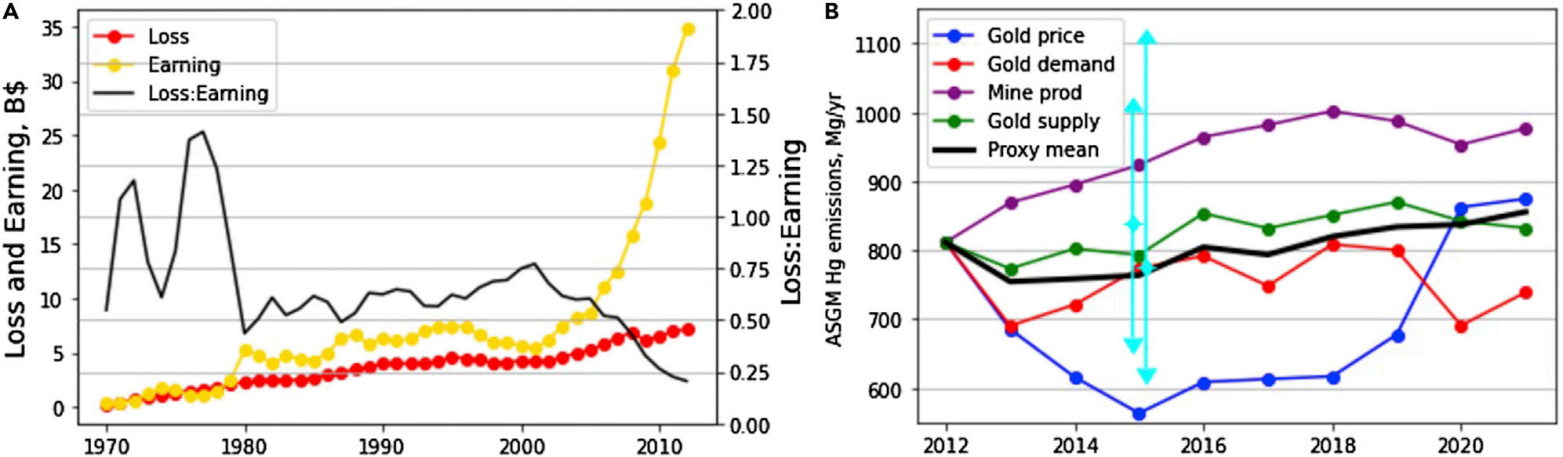
Trends of the health impact of Hg from ASGM (A) Comparisons between the health impact and the gold production earning during 1970–2012. The magenta dashed line shows the Loss: Earning ratio in the same year. The earnings/losses for individual years are discounted to their value in 2020 using a discount ratio of 3%. (B) Estimated ASGM Hg emissions during 2013–2021 using four alternative proxies: gold price, gold demand, mine gold production, and gold supply (https://www.gold.org). The mean of the four proxies is the black dashed line. Cyan vertical bars are other estimates (including both best estimate and ranges) (UNEP, 2018; Streets et al., 2019).

The global total accumulated economic loss due to ASGM activities is estimated to be $154 billion during 1970–2012 (with a discount rate of 3%, Figure 3A). The estimated loss increased from $0.59 billion in 1970 (contributed by 8.5×10^4^ points IQ decrease and 119 FHA deaths) to $7.1 billion in 2012 (5.8×10^5^ points of IQ decrements and 1,430 deaths), with an average increasing rate of 6.1% yr^−1^. This reflects the increasing food MeHg exposure associated with ASGM sources (Figure 1). The continuous increasing of population (1.5% yr^−1^) and newborns (0.3% yr^−1^, https://population.un.org) and economic development (3.3% yr^−1^, https://data.worldbank.org) during this period also contribute to this trend (Figure S3). We estimate the Hg emissions from ASGM during 2013–2021 based on proxy data including gold price, gold production, and supply following Streets et al. (2019) (Figure 3B). The gold price has decreased by up to 25% in the recent decade, suggesting a potentially lower profit for the miners. However, the gold production has been increasing in this period, likely to compensate for the lower price, which may suggest even more ASGM activities. The multi-proxy average shows a slightly increasing trend to 850 metric tons in 2021 with individual proxies indicating emissions ranging from 550 to 1000 metric tons in this period, consistent with the estimates by UNEP (2018) and Streets et al. (2019). This illustrates the persistent nature of the ASGM that needs action now, as required by the Minamata Convention (UNEP, 2017).

### Health risk comparison

The economic value of the health effects of the global general population caused by the ASGM source accounts for 48.3% of the total revenues (or earnings of miners) of the produced gold by this source (Figure 2B). The total accumulated value of ASGM gold production is $319 billion during 1970–2012 based on the annual production and gold price (Figure 3A). The revenue increased from $97 million in 1970 to $34.3 billion in 2012, much faster than the quantity of produced gold (86 tons in 1970 to 590 tons in 2012) due to the dramatical increase in the gold price, especially after 2005 (from ∼$35 oz^−1^ in 1970 to ∼$420 oz^−1^ in 2005 and ∼$1776 oz^−1^ in 2012, https://www.gold.org). The ratio of annual loss/earnings is higher in the first decade (50%–150%) due to the fast response of environmental Hg levels to ASGM emissions (Figures 1B–1D). The ratio is relatively stable (60%–70%) during 1980–2005, slightly varying following the gold price. The ratio has drastically decreased during 2005–2012 (to ∼20% in 2012), reflecting the fast increase in gold price (Figure 3A). This is indeed a concern for Hg emissions as the mining activity is even more profitable and may attract more miners. We expect the trend of ratio to level off in the recent decade, reflecting the stabilization in gold price (Figure 3B).

The economic value of the annual health impact on the global general population caused by the ASGM source ($7.1 billion) accounts for 6.0% of the global total health impact associated with MeHg exposure ($117 billion) calculated by Zhang et al. (2021) with a similar methodology. This fraction is indeed smaller than the contribution of ASGM source to the total Hg emissions at present day (37%). It is because of the relatively short history of ASGM emissions compared with other sources such as coal-fired power plants and cement production (Figure 1). This fraction also reflects a compound effect of the contributions of ASGM sources to multiple environmental compartments (atmospheric deposition, plankton, and soil) and food categories. The dose-response relationships between MeHg exposure and its health risk are not necessarily linear (see STAR Methods). This has very important implications for making Hg emission control policies: we need to consider the historical trends of emissions to attribute the Hg risk to different sectors and sources.

We find that the health risk imposed on the global general population is ∼1.5 times the risk to the ASGM miners and communities themselves. There has been no global-scale quantitative estimate for the risk so far, but they have been long identified as one of the highest impacted population groups, among other ones such as indigenous peoples, Arctic population, fish consumers, dental workers, and those living nearby contaminated sites (Basu et al., 2018). The ASGM miners (15 million) and the nearby communities (assuming a similar population size as miners, Weinhouse et al., 2021) account for 0.4% of the global total population. The median urine, blood, and hair Hg levels in ASGM population groups are typically ∼10 times higher than the general populations (Gibb and O’Leary, 2014; Basu et al., 2018). A rough first-order estimate shows that the magnitude of the risk imposed on this population group is ∼4% of the global total MeHg exposure risk of the general population caused by all sources (Zhang et al., 2021). This finding confirms the importance to consider not only the local impact but also the global effect associated with ASGM.

### Policy implications

Our study finds significant externality for ASGM by quantifying its global impact and reveals a major spatial decoupling between the health impact (mainly in the Global North) and its revenue (mainly in the Global South) (Figure 2). These findings help to create an economic incentive for the Global North to provide financial, technical, and institutional support and intervention. These interventions could focus on awareness-raising, capacity-building, and technology transfer in ASGM communities and countries, which have been proven effective by many pilot programs across South America, Southeast Asia, and Sub-Saharan Africa (e.g. Veiga and Chouinard, 2008; Spiegel and Veiga, 2010; Cordy et al., 2013). However, the scale of these measures as well as the spatial and temporal coverage is often limited by the availability of funds (Chen et al., 2018). A global estimate for the exact fund needs is still absent from the literature. Previous studies found that equipping each miner with a low-cost retort that effectively reduces Hg use costs ∼$300 million (average cost of $20 per retort) (Sippl and Selin, 2012), which is a lower-end estimate but is two to three orders of magnitudes smaller than the global health impact caused by ASGM. Indeed, the health impact caused by ASGM has been largely perceived as a local problem only for the miners and the nearby communities in the mining countries. Our results suggest that ASGM is also a global problem that needs a globally coordinated strategy, and the international intervention is highly cost-effective.

Synergies could be created between international financial help and other policies or measures. For instance, the ban on Hg mining and restrictions on Hg trade increases Hg prices, which potentially encourages the miners to adopt Hg-free mining techniques (Davis, 2014). National governments that receive the finical help are also responsible for the legal, political, and administrative issues as part of the National Action Plan (UNEP, 2017). Market-based mechanisms such as commodity certification can also create an incentive for environment-responsible miners and buyers (especially regarding jewelry choices) (Sippl and Selin, 2012). Indeed, curbing the Hg emission from ASGM sources is challenging, and the ongoing COVID-19 also hits ASGM communities heavily (Telmer and Kroll, 2020). The Minamata Convention has been focusing on individual countries developing and implementing their national action plans. Our study further highlights the importance of international assistance, which benefits both global health and local livelihoods.

### Limitations of the study

A limitation of our study is that the historical trend of our estimation of the health risk associated with ASGM may be subject to substantial uncertainties. The reported ASGM Hg emissions have increased from 351 metric tons in 2005 and 727 metric tons in 2010 to 838 tons in 2015 (UNEP, 2018; AMAP/UNEP, 2008; UNEP, 2013). Such a trend may be also driven by increased reporting and better data from many regions such as West Africa and South America (UNEP, 2013, 2018). Indeed, much of the ASGM activity is unacknowledged, unregulated, or even illegal in many countries, and thus lacks reliable official statistics (Zolnikov, 2020; Smith, 2019). Moreover, the mercury-related health impact may last much longer than a year (as we consider here) in the form of re-emissions from the land and ocean surfaces, our calculations are thus just indicative and a lower-end estimation. Uncertainties also exist in our MeHg Mercury and Risk model. For the calculation of MeHg exposure from freshwater fish, there is indeed a long time lag for higher-trophic-level wild-caught fish, which could result in a smoother trend of risks than we calculated. In addition, uncertainties also affect the accuracy of the calculation of the changes in FHA death associated with ASGM due to the limit in epidemiological studies.

In summary, the accuracy of health risk calculated by our study is limited by uncertainties existing in both the ASGM Hg emission estimates and the mercury-related health impacts estimates. More sound data related to the historical ASGM activities worldwide and further studies on the re-emission of Hg from different environments as well as the health risks of MeHg exposure from food are warranted to better estimate the global health risk of ASGM.

## STAR★Methods

### Key resources table

**Table:** 

REAGENT or RESOURCE	SOURCE	IDENTIFIER
**Deposited data**		

Health risks associated with ASGM	This manuscript	https://doi.org/10.17632/79257m8c8k.1
Health risk comparison	This manuscript	https://doi.org/10.17632/79257m8c8k.1
ASGM population	This manuscript (Weinhouse et al., 2021)	https://doi.org/10.17632/79257m8c8k.1

**Software and algorithms**		

MITgcm	This manuscript (Zhang et al., 2021)	https://doi.org/10.17632/79257m8c8k.1 https://github.com/MITgcm/MITgcm.git
GEOS-Chem	This manuscript (Zhang et al., 2021) (Horowitz et al., 2017)	https://doi.org/10.17632/79257m8c8k.1 https://www.geos-chem.seas.harvard.edu
GTMM	This manuscript (Smith-Downey et al., 2010)	https://doi.org/10.17632/79257m8c8k.1 https://www.geos-chem.seas.harvard.edu
NJUCPL	This manuscript (Zhang et al., 2019)	https://doi.org/10.17632/79257m8c8k.1
MeHg Mercury and Risk Model	This manuscript	https://doi.org/10.17632/79257m8c8k.1

### Resource availability

#### Lead contact

Further information and requests for resources should be directed to and will be fulfilled by the lead contact, Yanxu Zhang (zhangyx@nju.edu.cn).

#### Materials availability

This study did not generate new unique reagents.

### Method details

#### Mercury transport model

We employ a coupled online Earth system model framework to simulate the transport and fate of Hg emitted during ASGM following Zhang et al. (2021). The framework consists of a 3D atmospheric model (GEOS-Chem), a 3D ocean model (MITgcm), and a 2D terrestrial model (GTMM). These models are briefly described here and more details are available in Zhang et al. (2021) and references therein.

The atmospheric transport, dry and wet deposition, and redox chemistry of Hg are simulated by the GEOS-Chem model (version: v9-02) (Horowitz et al., 2017). The model is driven by GEOS-5 meteorological fields with a horizontal resolution of 4° latitude × 5° longitude and 47 vertical layers. The model simulates three tracers of Hg: gaseous elemental (Hg^0^), gaseous oxidized (Hg^II^), and particulate-bound divalent (Hg^P^). The redox chemistry of Hg includes oxidation of elemental Hg by bromine atoms and photoreduction of oxidized Hg in cloud droplets (Horowitz et al., 2017).

The fate of Hg in the terrestrial ecosystem and the soil and the land-atmosphere exchange is simulated by the GTMM (Global Terrestrial Mercury Model) (Smith-Downey et al., 2010). The model has a resolution of 1 × 1° and covers the top 30 cm of soil as a single layer. The model considers four soil Hg pools (fast, intermediate, slow, and armored) that are tied to carbon pools with different lifetimes against microbial respiration. The model takes atmospheric deposition as input and considers the bound of Hg to different soil carbon pools. The deposited Hg is also released back to the atmosphere after the soil carbon is respired by microbes.

We use the MITgcm to simulate the transport, chemistry, and trophic transfer of Hg in the ocean (Zhang et al., 2020). The model has a horizontal resolution of ∼1°x1°and 50 vertical levels. The ocean’s physical conditions are from the ECCO v4 state estimate (Forget et al., 2015). The model simulates tracers including elemental and oxidized Hg, methylmercury (mono- and di-), and Hg in plankton (six phytoplankton groups and two zooplankton types). The model considers photo- and biological mediated redox reactions of inorganic Hg and its methylation. The bioaccumulation and biomagnification of methylmercury in the marine plankton food web are also included. The ocean biogeochemical parameters are from an ocean plankton ecosystem model (Darwin) (Dutkiewicz et al., 2009).

The three models are coupled online to exchange Hg between components with an hourly time step using the NJUCPL (Zhang et al., 2019). The atmospheric deposition fluxes from GEOS-Chem are input to GTMM and MITgcm, and the re-emission fluxes from the latter two are input to the former. In the ASGM scenario, the three models are run from 1970 to 2012 with zero initial concentrations. The ASGM Hg emissions are used to drive the GEOS-Chem model, and the emissions are from the database for Global Atmospheric Research (EDGAR, version: v4.tox2, available during 1970–2012, https://edgar.jrc.ec.europa.eu). In the baseline scenario, we consider all the natural and anthropogenic emissions following Zhang et al. (2016). The initial condition of the atmosphere, land, and ocean are taken from previous simulations for the present day (Horowitz et al., 2017; Smith-Downey et al., 2010; Zhang et al., 2014).

#### MeHg Mercury and Risk model

The MeHg exposure for the general populations in individual countries is taken from Zhang et al. (2021), which was calculated based on literature-collected food MeHg concentrations and a food intake inventory for each country based on the database of the Food and Agriculture Organization of the United Nations (UN FAO, https://www.fao.org). The country-specific total MeHg exposure (*E*) was calculated as the sum of the product of MeHg concentrations (*C*) and food intake (*I*) for each food category (seafood, freshwater fish, and rice) (Equation 1). The different MeHg concentrations among trophic levels of fish/aquatic animals and the produced regions of rice were also considered.

(Equation 1) $$E_{\text{baseline}}=I_{\text{seafood}}C_{\text{seafood}}+I_{\text{fwfish}}C_{\text{fwfish}}+I_{\text{rice}}C_{\text{rice}}$$

The contribution of ASGM sources to the MeHg exposure is diagnosed by the ratio of the simulated environmental Hg levels between the ASGM and baseline scenarios. The contributions of ASGM sources to the MeHg concentrations in seafood, freshwater fish, and rice are diagnosed by the ratios of plankton MeHg concentration (*P*), atmospheric deposition (*D*), and total soil Hg concentrations (*S*), respectively, following Zhang et al. (2021):

(Equation 2) $$E_{\text{ASGM}}=\frac{P_{ASGM}}{P_{baseline}}I_{\text{seafood}}C_{\text{seafood}}+\frac{D_{\text{ASGM}}}{D_{\text{baseline}}}I_{\text{fwfish}}C_{\text{fwfish}}+\frac{S_{\text{ASGM}}}{{\text{S}}_{\text{baseline}}}I_{\text{rice}}C_{\text{rice}}$$

where fwfish stands for freshwater fish. This effectively assumes that the food MeHg concentrations respond linearly to the level of Hg added to the ecosystem, which is an upper limit or a conservative estimation of the ASGM contributions. Indeed, an ecosystem-level experimental study reveals a concaved curve in fish MeHg concentrations responding to the added Hg that is linearly increasing in a 15-year course (Blanchfield et al., 2022). This can be improved when more data are available in the future. We consider no time lags among environmental levels, food concentrations, and human exposure, which might be acceptable as most of the food items (e.g. rice, aqua- and mariculture) are harvested and consumed within a few years. The ecosystem-level study also shows a timely response of fish MeHg to the addition of Hg to the environment (Blanchfield et al., 2022).

Two health endpoints are considered for MeHg exposure in this study: decrease in IQ of newborns and FHA, following Zhang et al. (2021). A linear relationship without a threshold dose-response relationship is used for the IQ endpoint (Giang and Selin, 2016; Rice et al., 2010):

(Equation 3) ΔIQ = γλβ×ΔE×BW

where ΔIQ is the decrease of IQ in points, ΔE is the change in daily intake of MeHg from food, and BW is the body weight. The coefficients *β* transfers food exposure to blood Hg concentration, which is further transferred by *λ* to hair Hg concentrations. The coefficient *γ* represents the dose-effect relationship between hair Hg concentrations and IQ decrement. A log-linear relationship for the FHA effect is considered in this study (Rice et al., 2010):

(Equation 4) $$\Delta \text{CF }=\;\sum_{g}{\text{POP}}_{g}\times {\text{Cf}}_{g}\times \omega \times \left(1-e^{-\varphi \lambda \beta \times \Delta \text{E}\times \text{BW}}\right)$$

where ΔCF is the changes in FHA death, POP_g_ and Cf_g_ are the population and baseline FHA incidence rate of gender *g* (male and female), respectively. The *φ* is the dose-effect coefficient between hair Hg concentration and FHA risks. Due to the limit in epidemiological studies, the subjective coefficient ω is introduced to represent its uncertainties. The values of these parameters and the related input data are taken from Zhang et al. (2021).

The economic valuation of the two health endpoints (*H*) is also calculated following Zhang et al. (2021):

(Equation 5) H=EL×ΔIQ + VSL×ΔCF

where EL is the lifelong earning loss due to IQ decrement, and a value of $18,832 (2008 value) per IQ point is adopted following Bellanger et al. (2013). VSL is the value of statistical life, and we use a value of $6.3 million (2005 value) following Giang and Selin (2016). The EL and VSL values for each country are also adjusted by the purchasing power parity adjusted gross domestic product per capita (https://tntcat.iiasa.ac.at/SspDb). For simplicity, we assign the lifelong health impact of the cohort of newborns to the year of emissions, and we consider no time lag between MeHg exposure and its health impact. A discount ratio of 3% is used to realize the economic loss and gold production from 1970–2012 to 2012 (Giang and Selin, 2016). The health impact that is attributed to ASGM sources is thus calculated as:

(Equation 6) $$H\left(E_{\text{ASGM}}\right)=H\left(E_{\text{baseline}}\right)-H\left(E_{\text{baseline}}-E_{\text{ASGM}}\right)$$

## Data Availability

• Data including the Health risks associated with ASGM, the Health risk comparison and the ASGM population generated or analyzed in this study are available at: https://doi.org/10.17632/79257m8c8k.1. • The GEOS-Chem and GTMM model code are available at: https://doi.org/10.17632/79257m8c8k.1 and https://www.geos-chem.seas.harvard.edu. The MITgcm model code is available on GitHub: https://github.com/MITgcm/MITgcm.git and at: https://doi.org/10.17632/79257m8c8k.1. The NJUCPL model and all other code and scripts are also available from Mendeley Data: https://doi.org/10.17632/79257m8c8k.1. • Any additional information required to reanalyze the data reported in this work paper is available from the Lead contact upon request.
