# Supplementary material for: Global health impact of atmospheric mercury emissions from artisanal and small-scale gold mining
Source: iScience. 2022 Aug 4;25(9):104881. doi: 10.1016/j.isci.2022.104881 (PMC9418800; doi:10.1016/j.isci.2022.104881)
Supplement: Document S1. Figures S1–S4 [file mmc1.pdf]

iScience, Volume *25*

Supplemental information

Global health impact of atmospheric mercury emissions from artisanal and small-scale gold mining

Qiaotong Pang, Jing Gu, Haikun Wang, and Yanxu Zhang

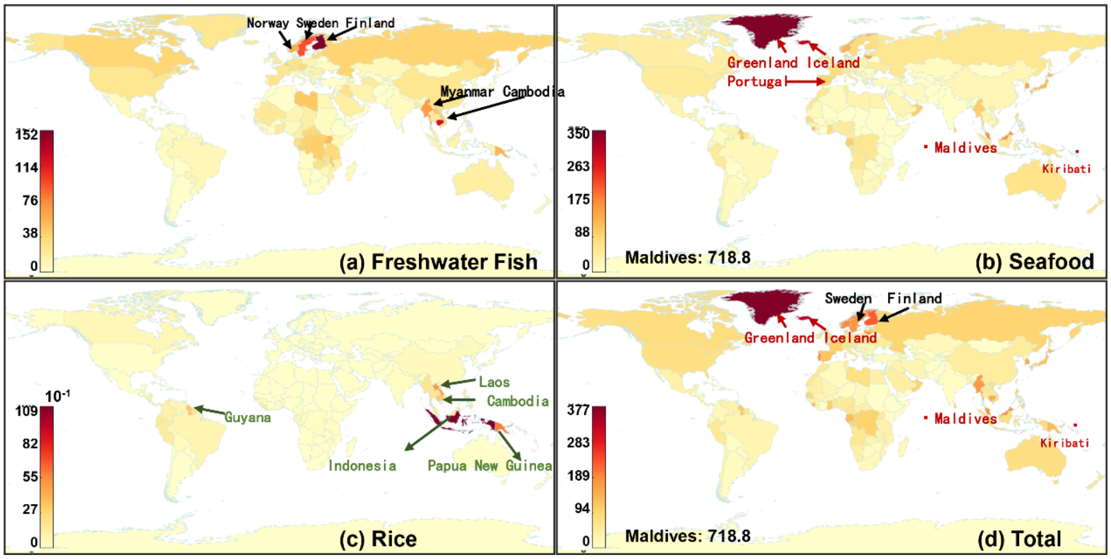

Fig. S1. ASGM-associated MeHg exposure for general populations from different food categories for individual countries at present-day. a) freshwater fish, b) seafood, c) rice, and d) the total of the above three, related to STAR Methods.

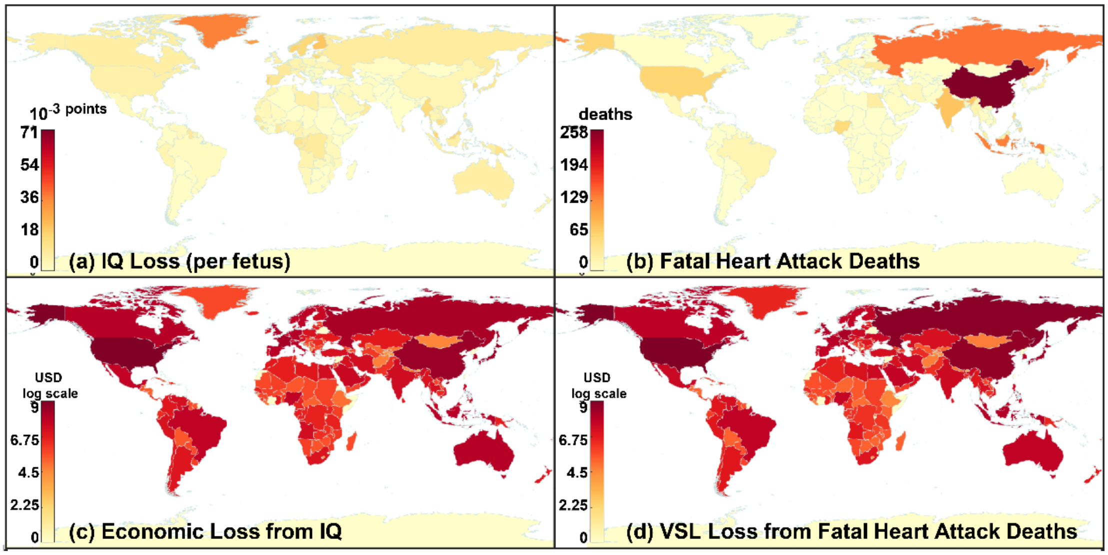

Fig. S2. Health risk from MeHg exposure associated with ASGM sources for general populations at present-day. a) IQ loss per fetus; b) fatal heart attack deaths; c) economic loss from IQ decrease; and d) value of statistical life loss from fatal heart attack deaths, related to Figure 2.

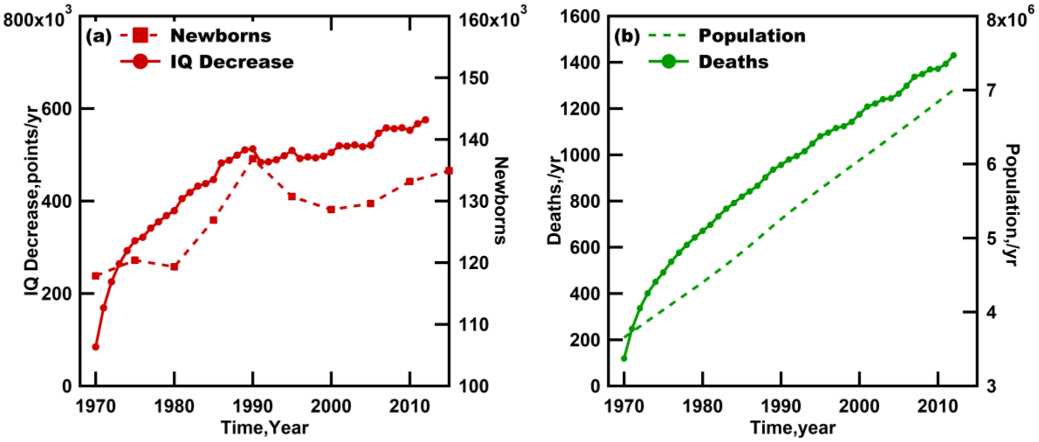

Fig. S3. Time trends of ASGM-related health risks due to MeHg exposure for global general populations. a) total IQ decreases (red solid line) and the number of newborns (red dashed line); b) total fatal heart attack deaths (green solid line) and global population (green dashed line), related to STAR Methods.

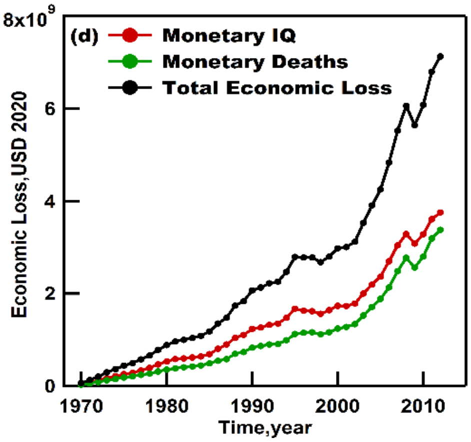

Figure S4. Historical economical valuation of the global health impact caused by the Hg emissions from ASGM. The red and green lines show the two endpoints: newborn IQ loss and fatal heart attack in adults, respectively. The black line shows the total of the two endpoints, related to Figure 3.
